# Ferric Carboxymaltose-Mediated Attenuation of Doxorubicin-Induced Cardiotoxicity in an Iron Deficiency Rat Model

**DOI:** 10.1155/2014/570241

**Published:** 2014-04-30

**Authors:** Jorge Eduardo Toblli, Carlos Rivas, Gabriel Cao, Jorge Fernando Giani, Felix Funk, Lee Mizzen, Fernando Pablo Dominici

**Affiliations:** ^1^Hospital Alemán, Laboratory of Experimental Medicine, School of Medicine, Avenue Pueyrredon 1640, 1118 Buenos Aires, Argentina; ^2^Instituto de Química y Fisicoquímica Biológica (UBA-CONICET), Facultad de Farmacia y Bioquímica, University of Buenos Aires, Buenos Aires, Argentina; ^3^Vifor (International) AG, Rechenstrasse 37, 9014 St. Gallen, Switzerland; ^4^Vifor Pharma, 1203-4464 Markham Street, Victoria, BC, Canada V8Z 7X8

## Abstract

Since anthracycline-induced cardiotoxicity (AIC), a complication of anthracycline-based chemotherapies, is thought to involve iron, concerns exist about using iron for anaemia treatment in anthracycline-receiving cancer patients. This study evaluated how intravenous ferric carboxymaltose (FCM) modulates the influence of iron deficiency anaemia (IDA) and doxorubicin (3–5 mg per kg body weight [BW]) on oxidative/nitrosative stress, inflammation, and cardiorenal function in spontaneously hypertensive stroke-prone (SHR-SP) rats. FCM was given as repeated small or single total dose (15 mg iron per kg BW), either concurrent with or three days after doxorubicin. IDA (after dietary iron restriction) induced cardiac and renal oxidative stress (markers included malondialdehyde, catalase, Cu,Zn-superoxide dismutase, and glutathione peroxidase), nitrosative stress (inducible nitric oxide synthase and nitrotyrosine), inflammation (tumour necrosis factor-alpha and interleukin-6), and functional/morphological abnormalities (left ventricle end-diastolic and end-systolic diameter, fractional shortening, density of cardiomyocytes and capillaries, caveolin-1 expression, creatinine clearance, and urine neutrophil gelatinase-associated lipocalin) that were aggravated by doxorubicin. Notably, iron treatment with FCM did not exacerbate but attenuated the cardiorenal effects of IDA and doxorubicin independent of the iron dosing regimen. The results of this model suggest that intravenous FCM can be used concomitantly with an anthracycline-based chemotherapy without increasing signs of AIC.

## 1. Introduction

Cytotoxic cancer treatments frequently include anthracyclines [1]. However, their use is associated with a risk of anthracycline-induced cardiotoxicity (AIC) leading to acute transient arrhythmias, chronic cardiomyopathy, and heart failure [2]. It is hypothesised that AIC results from anthracycline-mediated radical reactions and production of reactive oxygen species (ROS), which may involve anthracycline-iron complexes [3–5].

Considering that anaemia and iron deficiency (ID) are frequent complications in cancer patients [6, 7] and require iron supplementation [8, 9], clinicians are concerned that concomitant use of anthracyclines and iron might increase AIC. However, anaemia and ID should be treated since anaemia is associated with fatigue and impaired response to cancer treatment and survival [10–12]. Furthermore, even ID alone can increase oxidative/nitrosative stress in the heart [13]. Among available iron treatments, oral iron salts are often poorly tolerated whereas intravenous (i.v.) iron preparations are well tolerated at recommended doses and improve haematological response to erythropoiesis-stimulating agents [9, 14].

The primary objective of this study was to evaluate whether i.v. iron treatment (ferric carboxymaltose, FCM, Vifor Pharma) modulates doxorubicin- (DOX-) induced AIC. For this purpose, we established a model of DOX-induced cardiotoxicity in spontaneously hypertensive stroke-prone (SHR-SP) rats with iron deficiency anaemia (IDA). Since DOX is also nephrotoxic [15, 16], kidney assessments were performed in parallel (see Supplementary Material available online at http://dx.doi.org/10.1155/2014/570241).

## 2. Materials and Methods

### 2.1. Animals

All experiments were approved by the Hospital Aleman Animal Care and the Teaching and Research Committee and performed in accordance with the NIH Guide for the Care and Use of Laboratory Animals. Three-week-old male SHR-SP rats (Charles River Laboratories, USA) were acclimatized in a temperature-controlled room (22 ± 2°C) with free access to tap water and normal diet (iron content 250 ppm, Provimi-Kliba, Switzerland)* ad libitum*. During the study, animals received normal diet (ND) or low iron diet (LID, 7 ppm iron) as specified in Table 1. DOX was given as intraperitoneal (i.p.) and FCM as i.v. injection (both 0.5 mL).

### 2.2. Treatment

#### 2.2.1. Model 1

After 4 weeks of acclimatization, 48 SHR-SP rats were randomized into six groups (*n* = 8/group). Groups A–D were kept on LID for the entire study while Groups E-F received ND. After 12 weeks, Groups A–C received 6 weekly DOX injections of 3, 4, or 5 mg/kg BW, respectively. Group E received 6 weekly DOX injections of 4 mg/kg BW. Groups D and F received saline solution.

#### 2.2.2. Model 2

After 3 weeks of acclimatization, 64 SHR-SP rats were randomized into eight groups (*n* = 8/group). Groups 1–6 were fed with LID while Groups 7 and 8 were fed with ND. After 7 weeks, Groups 1–5 and Group 8 received 6 weekly DOX injections at 4 mg/kg BW. In addition, starting at the second weekly DOX injection, Groups 1 and 2 received 5 weekly FCM injections at 3 mg iron/kg BW concurrent with or 3 days after DOX treatment, respectively, while Groups 3 and 4 received a single FCM injection at 15 mg iron/kg BW concurrent with or 3 days after the second DOX injection, respectively. Group 8 received a single injection of FCM at 15 mg iron/kg BW concurrent with the second DOX injection. Group 5 received saline injections concurrent with DOX and Groups 6 and 7 received 6 weekly i.p. and 5 weekly i.v. saline injections, respectively.

Two weeks after the last DOX or saline injection (end of study), anaesthetized rats were euthanized by subtotal exsanguination, and tissue samples were taken for histological assessments.

### 2.3. Blood Pressure and Echocardiography

At study end, systolic and diastolic blood pressure (SBP and DBP) were measured in nonanaesthetized rats as described [17]. Transthoracic echocardiograms and assessment of interventricular septum (IVS) and left ventricular posterior wall (LVPW) diastolic thickness, left ventricular end-diastolic and end-systolic diameter (LVDD, LVSD), and fractional shortening (FS) were performed as described previously [18].

### 2.4. Laboratory Parameters

Blood hemoglobin (Hb) and serum iron were measured using routine blood analyzers (Hb: HemoCue, Sweden; SYSMEX XT 1800i, Japan; iron: Roche Diagnostics Autoanalyser Modular-P800, Germany). Serum transferrin was determined by radial immunodiffusion (Biocientifica, Argentina) and transferrin saturation (TSAT) was calculated as described previously [19]. Urine neutrophil gelatinase-associated lipocalin (NGAL) was measured by ELISA (BioPorto Diagnostics, Denmark).

### 2.5. Biochemical Assessment of Oxidative Stress in Heart Samples

Thiobarbituric acid reactive substances (TBARS), malondialdehyde (MDA), and glutathione, as well as the activities of catalase, Cu,Zn superoxide dismutase (Cu,Zn-SOD), and glutathione peroxidase (GPx) were quantified in tissue homogenates of the saline-perfused heart (left ventricular free wall [LVFW], left ventricular septum [LVS], and right ventricle [RV]) as previously described [20–23].

### 2.6. Histology

Three-micron sections of LVFW, LVS, and RV were fixed in phosphate-buffered, 10% formaldehyde (pH = 7.2), embedded in paraffin, and stained with Sirius Red [24]. Histomorphometric evaluations (width and density of cardiomyocytes, density of capillaries (vessels <8 *μ*m), and ratio cardiomyocytes/capillaries) were performed at ×400 magnification using a Nikon E400 light microscope and an image analyser (Image-Pro Plus 4.5 for Windows, Media Cybernetics LP, USA).

Immunohistochemistry was performed with antibodies against tumour necrosis factor-alpha (TNF-*α*, AF-510-NA, R&D Systems, USA), interleukin- (IL-) 6 (sc-1265, Santa Cruz Biotechnology, USA), inducible nitric oxide synthase (iNOS, sc-651, Santa Cruz Biotechnology), nitrotyrosine (AB5411, Millipore, USA), caveolin-1 (sc-824, Santa Cruz Biotechnology), collagen type III (MU-167, Biogenex, USA), and ferritin light chain (L-ferritin, sc-14420, Santa Cruz Biotechnology). Staining was performed with a modified avidin-biotin-peroxidase complex and haematoxylin counterstaining as described [25].

All histological stainings are expressed as percentage of positive staining per area from 20 random images that were viewed and evaluated independently by two investigators who were blinded to sample identity.

### 2.7. Statistical Methods

Data are shown as mean ± standard deviation (SD). Statistical analyses used absolute values and GraphPad Prism 5.01 for Windows (GraphPad Software, USA). Normally distributed parameters were compared among groups using ANOVA. Parameters with non-Gaussian distribution (e.g., histological data) were compared using Kruskal-Wallis test (nonparametric ANOVA) and Dunn's multiple comparison test.

## 3. Results

### 3.1. Model 1: DOX-Induced Cardiotoxicity in SHR-SP Rats on Low Iron Diet (LID)

#### 3.1.1. Anaemia and Iron Status

LID-fed rats developed IDA as indicated by reduced Hb and TSAT (Groups D versus F; *P* < 0.05; Table 1). In LID-fed rats treated with DOX, Hb but not TSAT was further reduced compared to LID alone (Groups A–C versus D; *P* < 0.05). DOX-treated rats on ND had lower Hb levels than untreated rats (Groups E versus F; *P* < 0.05).

#### 3.1.2. Cardiovascular Function

As previously described in SHR-SP rats [26], systolic and diastolic blood pressure (SBP and DBP) increased in all groups during the study, with similar end of study values, except for lower values in LID-fed rats (Groups D versus A–C, E, and F; *P* < 0.01). Based on echocardiography, LID-fed rats exhibited increased left ventricle dimensions (LVDD and LVSD) and decreased FS compared to ND-fed rats (Groups D versus F; *P* < 0.05; Table 1). These cardiac alterations were aggravated by DOX treatment in a dose-dependent fashion (Groups A–C and E versus F; *P* < 0.05). Similar trends were observed for the marker of cardiac damage, caveolin-1.

#### 3.1.3. Oxidative/Nitrosative Stress and Inflammation

In the heart of LID-fed rats (Group D), markers of oxidative stress (Cu,Zn-SOD, GPx, and MDA), nitrosative stress (nitrotyrosine), and inflammation (TNF-*α*) were significantly elevated compared to those of ND-fed animals (Group F; Table 1). DOX treatment increased tissue levels of these markers in LID-fed rats in a dose-dependent manner (Groups A–C) compared to LID alone (Group D). As expected, the reduced to oxidised glutathione ratio (GSH : GSSG ratio) followed an inverse pattern.

#### 3.1.4. Mortality

In contrast to LID alone, the combination of LID and DOX resulted in a dose-dependent increase of mortality in SHR-SP rats; 13%, 25%, and 50% for DOX doses of 3, 4, and 5 mg/kg, respectively.

### 3.2. Model 2: Modulation of DOX-Induced Cardiotoxicity with FCM

#### 3.2.1. Anaemia and Iron Status

Hb, serum iron, and TSAT as well as L-ferritin in the heart were significantly reduced in LID-fed rats compared to ND-fed rats (Groups 6 versus 7; *P* < 0.01; Table 2), and Hb was further decreased in rats receiving DOX (Group 5; *P* < 0.01). In DOX-treated, LID-fed rats, all FCM regimens resulted in significantly higher Hb, serum iron, TSAT, and L-ferritin compared to saline treatment (Groups 1–4 versus 5; *P* < 0.01). Identical trends were observed for L-ferritin immunostaining in the kidney (Table S3). Comparing FCM regimens, a single dose of concomitant or 3-day delayed FCM (15 mg iron/kg) after the second weekly DOX injection resulted in slightly higher Hb levels at study end than five weekly concomitant or delayed FCM doses (each 3 mg iron/kg; Groups 3 and 4 versus 1 and 2, respectively; *P* < 0.05).

#### 3.2.2. Blood Pressure and Echocardiography

The increase in mean blood pressure from baseline (SBP 160–167 mm Hg and DBP 93-94 mm Hg) to end of study (SBP 194–205 mm Hg and DBP 120–123 mm Hg) was similar in all treatment groups.

Echocardiography indicated that LID-fed rats had increased LVDD, LVSD, and LV mass (LVM) and decreased FS compared to ND-fed (Groups 6 versus 7; *P* < 0.01; Table 3). Addition of DOX to LID exacerbated all of these cardiac abnormalities compared to LID alone (Groups 5 versus 6; *P* < 0.01). In DOX-treated, LID-fed rats, FCM significantly improved these LV structural and functional abnormalities compared to saline (Groups 1–4 versus 5; *P* < 0.01). Among FCM regimens, LVSD improvements were greater following a single FCM dose than five weekly FCM doses (Groups 3 and 4 versus 1 and 2; *P* < 0.01). Similarly, FCM treatment significantly improved proteinuria and urine NGAL levels (Table S1). Creatinine clearance was not affected by DOX in LID-fed rats. Among FCM-treated groups, NGAL levels were significantly lower in rats given a single FCM dose three days after DOX compared to other FCM regimens (Groups 4 versus 1–3; *P* < 0.01).

#### 3.2.3. Oxidative/Nitrosative Stress and Inflammation

**Figure 1:**
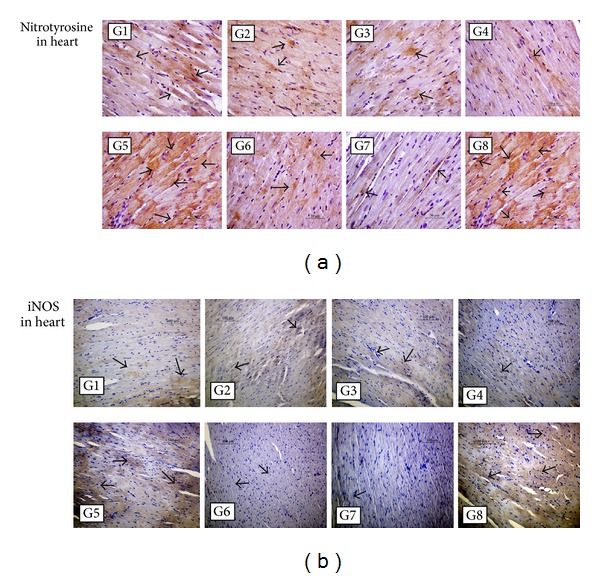
Immunohistochemical detection of nitrosative stress in the heart. Light micrographs of heart sections from rats treated as outlined in Materials and Methods Section and Table 2. In LID-fed rats (Groups 1–6 (G1–G6)), DOX treatment (G5) increased positive immunostaining (arrows) for nitrotyrosine and inducible nitric oxide synthase (iNOS) compared to saline treatment (G6), whereas FCM administration to DOX-treated rats (G1–G4) reduced levels of nitrotyrosine and iNOS compared to DOX treatment alone (G5). In ND-fed rats (G7 and G8), treatment with DOX and FCM (G8) greatly increased positive immunostaining compared to ND alone (G7). (a) Original magnification, ×400. (b) Original magnification, ×200.

**Figure 2:**
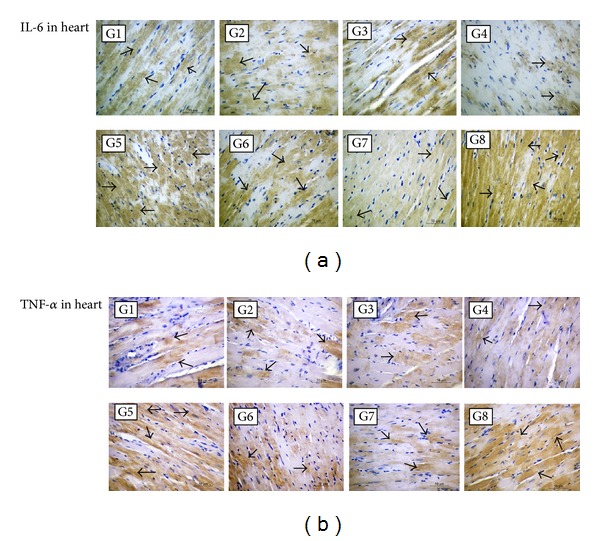
Immunohistochemical detection of inflammation in the heart. Light micrographs of heart sections from rats treated as outlined in Materials and Methods Section and Table 2. In LID-fed rats (Groups 1–6 (G1–G6)), DOX treatment (G5) increased positive immunostaining (arrows) for interleukin-6 (IL-6) and tumour necrosis factor-*α* (TNF-*α*) compared to saline treatment (G6), whereas FCM administration to DOX-treated rats (G1–G4) reduced levels of IL-6 and TNF-*α* compared to DOX treatment alone (G5). For IL-6, FCM treatment reduced staining levels below that of LID alone (G6). In ND-fed rats (G7 and G8), treatment with DOX and FCM (G8) greatly increased positive immunostaining compared to ND alone (G7). (a) and (b) original magnification, ×400.

Markers of oxidative stress (TBARS, Cu,Zn-SOD, GPx, and GSH : GSSG ratio), nitrosative stress (nitrotyrosine and iNOS), inflammation (TNF-*α* and IL-6), and fibrosis (collagen III) in the heart were increased in LID-fed compared to ND-fed rats (Groups 6 versus 7; *P* < 0.01; Table 4; Figures 1 and 2). DOX treatment further increased these stress and inflammation-related markers in LID-fed rats compared to saline (Groups 5 versus 6; *P* < 0.01). In DOX-treated, LID-fed rats, FCM significantly reduced oxidative stress (Groups 1–4 versus 5; *P* < 0.01). Among FCM treatments, significantly lower levels of oxidative stress were observed with a single FCM dose compared to five weekly FCM doses for all markers except GPx (Groups 3 and 4 versus 1 and 2; *P* < 0.01). Similarly, oxidative/nitrosative stress and proinflammatory markers in kidneys of LID-fed rats were increased compared to ND-fed. Further increase by DOX treatment was at least partly recovered by FCM treatment (Tables S2 and S3 and Figures S1 and S2). As in the cardiac assessment, greater reductions in renal oxidative stress markers were seen with a single FCM dose compared to the repeated dosing (Groups 3 and 4 versus 1 and 2; *P* < 0.01).

#### 3.2.4. Histological Evaluation

Increased extracellular matrix expansion and interstitial fibrosis was seen in LID-fed compared to ND-fed rats (Groups 6 versus 7; Table 5). The DOX-associated further increase of fibrosis in LID-fed rats (Groups 5 versus 6; *P* < 0.01) was largely normalized with all FCM regimens (Groups 1–4 versus 5; *P* < 0.01). Enlargement of cardiomyocytes was observed in LID-fed compared to ND-fed rats (Groups 6 versus 7) and was aggravated by DOX (Groups 5 versus 6; *P* < 0.01). All tested FCM treatments in LID-fed rats significantly improved cardiomyocyte and capillary morphometric measures compared to saline (Groups 1–4 versus 5; *P* < 0.01). In the kidney, histological assessment showed similar trends (Table S3).

#### 3.2.5. Effects of FCM Treatment in Iron-Replete Rats

ND-fed rats treated with DOX and a single dose of FCM on the same day as the second weekly DOX treatment (Group 8) had higher levels of serum iron and TSAT compared to LID-fed rats given the same DOX and FCM treatments (Group 3; Table 2). Also cardiac tissue L-ferritin levels as well as cardiac oxidative/nitrosative stress, proinflammatory, and fibrosis markers were increased (Tables 4 and 5; Figures 1 and 2), whereas cardiac function was reduced (Table 3). Kidney assessments showed similar results, that is, higher tissue L-ferritin levels (Table S3), as well as higher oxidative/nitrosative stress, proinflammatory (Tables S2 and S3; Figures S1 and S2), fibrosis (Table S3), and kidney damage markers (Table S1).

#### 3.2.6. Mortality

No deaths occurred in saline-treated, ND-fed rats (Group 7) or any LID-fed group regardless of treatment (Groups 1–6). Deaths were reported for two of eight ND-fed rats treated with concomitant DOX and FCM (15 mg iron/kg) (Group 8).

## 4. Discussion

The data presented herein show that FCM at a total dose of 15 mg iron/kg BW resolves IDA and attenuates IDA- and DOX-associated cardiorenal toxicity in SHR-SP rats. In line with prior clinical and nonclinical studies, IDA alone increased cardiac and renal oxidative/nitrosative stress and inflammation as well as abnormalities in cardiac morphology and function in the models described here [13, 27–29]. DOX treatment further increased oxidative/nitrosative stress and inflammation and exacerbated cardiorenal toxicity, whereas supplementation with FCM significantly improved these parameters.

The development of hypertension in SHR-SP rats [26] was not affected by LID, DOX treatment, or combined treatment with DOX and FCM.

Beneficial effects of FCM were observed whether administered as a single dose or as repeated 3 mg/kg doses; yet single dose administration was associated with a trend towards higher Hb as well as less oxidative stress, LVSD, and urinary NGAL (an early marker of acute kidney damage) [30]. Also administration of FCM either concurrent with or three days after DOX treatment was equally effective. The investigated regimens reflect the clinical setting in terms of total dose infusion and possible treatment schedules in DOX-treated patients.

Anthracycline-mediated production of ROS is considered an important mechanism of AIC, and iron loading has been suggested to potentiate this mechanism [31]. Therefore, there are concerns that concomitant administration of iron and anthracyclines could exacerbate AIC [9]. However, FCM did not increase AIC in this model. Moreover, the correction of IDA with FCM markedly attenuated AIC. There are interesting parallels between the pathological features of IDA- and DOX-associated cardiotoxicity, including left ventricular dilatation and hypertrophy that can progress to heart failure [2, 24, 27]. While the pathogenic mechanisms leading to these characteristic LV changes may differ between IDA and DOX treatment, it is noteworthy that perturbation of iron metabolism and elevation of oxidative stress/inflammation is common in both [32, 33]. Therefore, clinical data in iron-deficient chronic heart failure (CHF) patients showing that FCM improved functional status, quality of life, and exercise capacity [27, 34] are in line with the FCM-based improvement of IDA- and DOX-associated stress and cardiac impairment in SHR-SP rats.

In rodents, DOX-induced cardiotoxicity is increased by dietary and genetic iron overload [35, 36]. In the present study, combined treatment of iron-replete (ND-fed) SHR-SP rats with DOX and i.v. iron markedly increased cardiorenal toxicity. Although the relative contributions of the two treatments cannot be determined in our study, we hypothesise that excessive levels of systemic iron can exacerbate the cardiorenal toxicity of DOX [37, 38].

Notably, AIC can occur as “acute” cardiotoxicity within hours or days of treatment as well as early-onset or late-onset AIC within one year or after one year of treatment, respectively [2]. Acute AIC often presents as disturbances in intracardiac conduction and arrhythmias, whereas early- and late-onset cardiotoxicity are associated with LV dysfunction and can lead to heart failure. Our model system investigated the modulation of acute AIC and showed that, in iron-deficient hypertensive rats, concomitant i.v. iron can maintain normal contractility of the heart. Whether the reduction or prevention of acute AIC has also a positive influence on early- or late-onset AIC has to be investigated in a model with a longer follow-up period.

Since this study focused on the characterisation of DOX-induced cardiotoxicity and its modulation with i.v. iron, rats were nontumour bearing. However, comorbid hypertension was addressed by using SHR-SP rats. Dietary iron restriction resulted in IDA as well as significant oxidative stress and inflammation, which were aggravated by DOX. The decrease in Hb associated with DOX alone suggests that iron-independent effects on erythropoiesis, possibly mediated by oxidative stress and inflammation, are also addressed in this model. Hence, the model combines features of IDA and chemotherapy-induced anaemia. Nevertheless, the results of this study are based on a model of absolute iron deficiency and should not be extrapolated to patients with functional iron deficiency since the biodistribution and erythropoietic efficacy of i.v. iron may be different in the clinical setting of cancer-associated anaemia.

## 5. Conclusion

FCM did not aggravate DOX-induced cardiotoxicity in iron-deficient hypertensive rats receiving chronic DOX treatment. Moreover, administration of FCM attenuated cardiorenal oxidative/nitrosative stress, inflammation, and fibrosis as well as effects on cardiac and renal morphology and function compared to saline controls.

## Supplementary Material

DOX-associated nephrotoxicity and its modulation with FCM were characterised in SHR-SP rats with IDA that was induced by low iron diet. The assessments involved the following evaluations at study end: renal function (creatinine clearance, 24 h proteinuria, urine NGAL), oxidative stress (GSH:GSSG ratio, MDA, catalase, Cu,Zn-SOD, GPx activity), histological and immunohistochemical parameters (Masson's Trichrome and Sirius Red staining, immunodetection of TNF-alpha, IL-6, iNOS, nitrotyrosine and ferritin L-chain).

## Figures and Tables

**Table 1:** Haematological, functional, and oxidative/nitrosative stress and inflammatory markers according to iron diet and DOX treatment.

	Group					
	A	B	C	D	E	F
Parameter						
Iron diet	LID	LID	LID	LID	ND	ND
DOX dose (mg/kg BW)	3	4	5	0	4	0
Hb (g/dL)	8.9 ± 0.5	8.6 ± 0.4	8.3 ± 0.5	10.3 ± 0.5^†^	13.4 ± 0.7^‡^	17.1 ± 0.5*
TSAT (%)	13.8 ± 2.3	11.9 ± 9.0	10.9 ± 4.3	12.7 ± 2.0	36.4 ± 3.0^‡^	41.8 ± 4.1^‡^
LVDD (mm)	8.61 ± 0.12	8.70 ± 0.14^||^	9.02 ± 0.13*	8.30 ± 0.12	8.28 ± 0.14	7.80 ± 0.10*
LVSD (mm)	5.60 ± 0.11	6.00 ± 0.10^||^	6.34 ± 0.11*	5.10 ± 0.10	5.20 ± 0.11	4.50 ± 0.12*
FS (%)	34.9 ± 1.7	31.0 ± 1.3^||^	29.6 ± 1.4*	38.6 ± 1.4^†^	37.2 ± 1.6^†^	42.3 ± 1.9*
Caveolin-1 (%/area)	18.2 ± 2.0	15.6 ± 1.8^||^	14.9 ± 2.1^||^	23.1 ± 2.2^†^	24.7 ± 3.0^†^	32.3 ± 2.7*
Cu,Zn-SOD (U/mg protein)	33.5 ± 2.3	37.2 ± 2.6^||^	40.1 ± 2.8^§^	24.7 ± 1.9^†^	28.4 ± 2.1^†^	18.9 ± 1.5*
GPx (U/mg protein)	423.2 ± 22.7	460.5 ± 26.4^||^	488.4 ± 30.1^§^	376.9 ± 32.4^†^	389.4 ± 27.4^†^	264.3 ± 20.6*
MDA (nmol/mg protein)	3.4 ± 0.1	3.7 ± 0.2^||^	4.0 ± 0.2^§^	2.8 ± 0.3^†^	3.0 ± 0.2^†^	1.9 ± 0.2*
GSH : GSSG ratio	3.0 ± 0.2	2.7 ± 0.1^||^	2.3 ± 0.3^§^	4.0 ± 0.3^†^	3.8 ± 0.2^†^	4.7 ± 0.3*
Nitrotyrosine (%/area)	24.1 ± 3.2	28.9 ± 3.7^||^	34.8 ± 3.0^§^	14.9 ± 2.6^†^	16.7 ± 2.1^†^	12.2 ± 2.0*
TNF-*α* (%/area)	18.7 ± 2.0	19.2 ± 1.6	20.1 ± 2.3	14.8 ± 1.7^†^	15.1 ± 1.9^†^	12.1 ± 2.0*

Data presented as mean ± SD. LID: low iron diet; ND: normal diet; DOX: doxorubicin; Hb: hemoglobin; TSAT: transferrin saturation; LVDD: left ventricular end-diastolic diameter; LVSD: left ventricular end-systolic diameter; FS: fractional shortening; Cu,Zn-SOD: copper-zinc superoxide dismutase; GPx: glutathione peroxidase; MDA: malondialdehyde; GSH : GSSG: reduced glutathione : oxidised glutathione; TNF-*α*: tumour necrosis factor alpha.

*P < 0.05 versus all groups; ^†^ *P* < 0.05 versus A, B, and C; ^‡^ *P* < 0.01 versus A, B, C, and D; ^§^ *P* < 0.05 versus A and B; ^||^ *P* < 0.05 versus A.

**Table 2:** Anaemia and iron status according to iron diet, DOX treatment, and supplementation with FCM.

	Group							
	1	2	3	4	5	6	7	8
Treatment								
Iron diet	LID	LID	LID	LID	LID	LID	ND	ND
DOX (4 mg/kg BW)	+	+	+	+	+	−	−	+
FCM (mg iron/kg BW)	3	3	15	15	−	−	−	15
	Concurrent with DOX	Three days after DOX	Concurrent with DOX	Three days after DOX				Concurrent with DOX
Saline	−	−	−	−	+	+	+	−
Parameter								
Hb (g/dL)	14.5 ± 0.5^‡^	14.6 ± 0.3^‡^	15.6 ± 0.4	15.5 ± 0.6	10.3 ± 0.3^†§^	11.0 ± 0.3^†^	17.2 ± 0.7*	17.0 ± 0.7*
Serum iron (*μ*g/dL)	214.3 ± 17.7	218.7 ± 15.8	221.8 ± 16.0	223.6 ± 15.0	117.1 ± 14.0^†^	121.0 ± 15.4	248.6 ± 16.6^∗||^	320.4 ± 19.5^∗¶^
TSAT (%)	36.2 ± 2.1	36.3 ± 2.3	36.2 ± 2.0	36.4 ± 2.1	19.2 ± 3.1^†^	20.0 ± 3.2	41.3 ± 2.1^∗||^	47.8 ± 2.4^∗¶^
L-Ferritin (%/area)	10.3 ± 1.6	10.5 ± 1.0	10.4 ± 1.1	10.7 ± 1.4	1.8 ± 0.4^†^	1.6 ± 0.3^†^	3.1 ± 0.5*	15.7 ± 1.9*

Data presented as mean ± SD. LID: low iron diet; ND: normal diet; DOX: doxorubicin; FCM: ferric carboxymaltose; Hb: hemoglobin; TSAT: transferrin saturation.

*P < 0.05 for G7 and G8 versus G1, G2, G3, G4, G5, and G6; ^†^ *P* < 0.01 for G5 and G6 versus G1, G2, G3, and G4; ^‡^ *P* < 0.05 for G1 and G2 versus G3 and G4; ^§^ *P* < 0.01 for G5 versus G6; ^||^ *P* < 0.05 for G7 versus G1, G2, G3, and G4; ^¶^ *P* < 0.01 for G8 versus G1, G2, G3, G4, and G7.

**Table 3:** Echocardiographic outcomes according to iron diet, DOX treatment, and supplementation with FCM.

	Group							
	1	2	3	4	5	6	7	8
Parameter								
LVDD (mm)	8.00 ± 0.09^§^	8.01 ± 0.09^§^	7.91 ± 0.10^§§^	7.90 ± 0.10^§§^	8.55 ± 0.11^‡^	8.17 ± 0.08^†^	7.81 ± 0.10^||^	8.58 ± 0.11
LVSD (mm)	5.01 ± 0.08*	5.00 ± 0.09*	4.85 ± 0.09^§^	4.80 ± 0.10^§^	5.80 ± 0.10**	5.25 ± 0.08^†^	4.31 ± 0.11^||^	6.00 ± 0.10
LVPW (mm)	2.36 ± 0.10	2.33 ± 0.12	2.34 ± 0.11	2.33 ± 0.10	2.39 ± 0.12	2.36 ± 0.11	2.30 ± 0.12	2.29 ± 0.10
IVS (mm)	2.36 ± 0.10	2.36 ± 0.11	2.36 ± 0.09	2.36 ± 0.10	2.46 ± 0.11	2.39 ± 0.11	2.31 ± 0.10	2.30 ± 0.10
FS (%)	37.4 ± 0.5^||||^	37.6 ± 0.5^||||^	38.7 ± 0.6^§^	39.2 ± 0.9^§^	32.2 ± 1.0**	35.7 ± 0.6^†^	44.8 ± 1.1^||^	30.1 ± 1.3
LVM (g)	1.33 ± 0.06^¶^	1.31 ± 0.07^‡‡^	1.28 ± 0.09^‡‡^	1.25 ± 0.08^‡‡^	1.48 ± 0.06**	1.36 ± 0.06^††^	1.20 ± 0.07^||^	1.32 ± 0.08

Data presented as mean ± SD. A brief tabular overview of treatments in Groups 1–8 is included in Table 2. Further details are described in the Materials and Methods section. LID: low iron diet; ND: normal diet; DOX: doxorubicin; FCM: ferric carboxymaltose; LVDD: left ventricular end-diastolic diameter; LVSD: left ventricular end-systolic diameter; LVPW: left ventricular posterior wall thickness; IVS: interventricular septum thickness; FS: fractional shortening; LVM: left ventricular mass.

*P < 0.01 versus G3 to G8; ^†^ *P* < 0.01 versus G7 and G8; ^‡^ *P* < 0.01 versus G6 and G7; ^§^ *P* < 0.01 versus G5 to G8; ^||^ *P* < 0.01 versus G8; ^¶^ *P* < 0.01 versus G5 and G7; **P < 0.01 versus G6 to G8; ^††^ *P* < 0.01 versus G7; ^‡‡^ *P* < 0.01 versus G5; ^§§^ *P* < 0.01 versus G5, G6, and G8; ^||||^ *P* < 0.01 versus G4 to G8.

**Table 4:** Cardiac oxidative/nitrosative stress and inflammation according to iron diet, DOX treatment, and supplementation with FCM.

	Group							
	1	2	3	4	5	6	7	8
Parameter								
MDA (nmol/mg protein)	3.6 ± 0.2**	3.5 ± 0.3**	2.9 ± 0.1^††^	2.7 ± 0.2*	4.3 ± 0.2^‡‡^	2.5 ± 0.2^§§^	1.8 ± 0.2^||^	5.7 ± 0.3
Cu,Zn-SOD (U/mg protein)	36.9 ± 3.2**	34.9 ± 2.0**	30.4 ± 2.1^††^	27.3 ± 3.0*	42.3 ± 2.4^‡‡^	26.3 ± 2.1^§§^	16.0 ± 1.8^||^	49.2 ± 3.3
GPx (U/mg protein)	409 ± 24**	399 ± 20^‡‡^	360 ± 20^††^	353 ± 23*	459 ± 24^‡‡^	320 ± 24^§§^	256 ± 21^||^	507 ± 32
GSH : GSSG ratio	2.5 ± 0.2**	2.6 ± 0.1**	3.6 ± 0.2^††^	3.8 ± 0.2*	2.4 ± 0.2^‡‡^	3.9 ± 0.1^§§^	4.8 ± 0.2^||^	2.0 ± 0.2
Nitrotyrosine (%/area)	18.0 ± 3.3	17.1 ± 2.6	16.9 ± 3.1	16.1 ± 2.2*	29.1 ± 3.4^‡^	16.8 ± 3.8	11.6 ± 1.3^†^	34.5 ± 4.5^‡^
iNOS (%/area)	6.9 ± 1.0	6.4 ± 1.1	6.7 ± 0.8	6.5 ± 0.9	21.4 ± 1.7^‡^	2.5 ± 0.4^†^	2.3 ± 0.6^†^	20.3 ± 2.0^‡^
TNF-*α* (%/area)	15.1 ± 1.4	14.4 ± 1.5	14.2 ± 1.2	14.1 ± 1.3	19.6 ± 1.4^‡^	15.0 ± 1.5	11.9 ± 1.1^§^	23.6 ± 2.0^§^
IL-6 (%/area)	15.7 ± 2.2	14.6 ± 1.7	15.2 ± 1.4	14.4 ± 1.6	28.4 ± 2.5^‡^	20.3 ± 1.6^†^	3.6 ± 1.0^§^	34.9 ± 3.1^§^
Collagen III (%/area)	7.4 ± 1.0	7.5 ± 0.9	7.3 ± 1.1	6.6 ± 0.9	13.0 ± 1.5^¶^	7.0 ± 1.1	4.3 ± 0.7^§^	13.9 ± 1.9^¶^

Data presented as mean ± SD. A brief tabular overview of treatments in Groups 1–8 is included in Table 2. Further details are described in the Materials and Methods section. MDA: malondialdehyde; Cu,Zn-SOD: copper-zinc superoxide dismutase; GPx: glutathione peroxidase; GSH : GSSG: reduced glutathione : oxidised glutathione; iNOS: inducible nitric oxide synthase; TNF-*α*: tumour necrosis factor-alpha; IL-6: interleukin-6.

*P < 0.01 versus G5, G7, and G8; ^†^ *P* < 0.01 versus G1, G2, G3, and G4; ^‡^ *P* < 0.01 versus G1, G2, G3, G4, G6, and G7; ^§^ *P* < 0.01 versus all groups; ^||^ *P* < 0.01 versus G8; ^¶^ *P* < 0.01 versus G1, G2, G3, G4, and G6; **P < 0.01 versus G3 to G8; ^††^ *P* < 0.01 versus G5 to G8; ^‡‡^ *P* < 0.01 versus G6 to G8; ^§§^ *P* < 0.01 versus G7, G8.

**Table 5:** Histomorphometric assessment of the heart according to iron diet, DOX treatment, and supplementation with FCM.

	Group							
	1	2	3	4	5	6	7	8
Parameter								
Masson's trichrome (% area)	13.7 ± 1.1*	14.3 ± 1.4*	13.0 ± 1.2*	12.1 ± 1.4*	17.4 ± 0.9^‡^	11.7 ± 1.8^||^	8.1 ± 1.2^¶^	20.9 ± 1.2
Sirius Red (% area)	10.3 ± 1.1*	9.9 ± 1.4*	9.8 ± 1.3*	9.3 ± 1.1*	15.9 ± 1.1^‡^	11.0 ± 1.2^||^	7.8 ± 1.3^¶^	19.3 ± 1.8
Cardiomyocyte width (µm)	31.7 ± 1.1^†^	31.3 ± 1.2^†^	31.6 ± 1.1^†^	31.0 ± 1.0^†^	35.2 ± 0.9^‡^	33.4 ± 0.4^||^	31.1 ± 0.9^†^	31.4 ± 0.9^†^
Cardiomyocyte density (*n*/area)	26.7 ± 0.8^†^	27.1 ± 1.0^†^	26.8 ± 0.8^†^	27.2 ± 0.8^†^	20.1 ± 1.6^§^	25.2 ± 0.7	27.1 ± 0.7^†^	26.9 ± 0.8^†^
Capillary density (*n*/area)	18.5 ± 0.7^†^	19.0 ± 1.0^†^	18.8 ± 0.9^†^	19.1 ± 0.7^†^	12.3 ± 1.1^§^	17.1 ± 1.0	19.1 ± 1.3	18.9 ± 1.1
Cardiomyocyte/capillary ratio	0.71 ± 0.03^†^	0.71 ± 0.03^†^	0.70 ± 0.02^†^	0.70 ± 0.03^†^	0.61 ± 0.01^§^	0.67 ± 0.03	0.71 ± 0.03^†^	0.70 ± 0.04^†^

Data presented as mean ± SD. A brief tabular overview of treatments in Groups 1–8 is included in Table 2. Further details are described in the Materials and Methods Section.

*P < 0.01 versus G5, G6, G7, and G8; ^†^ *P* < 0.01 versus G5 and G6; ^‡^ *P* < 0.01 versus G6, G7, and G8; ^§^ *P* < 0.01 versus G6; ^||^ *P* < 0.01 versus G7 and G8; ^¶^ *P* < 0.01 versus G8.
