# Supplementary material for: Ferric Carboxymaltose-Mediated Attenuation of Doxorubicin-Induced Cardiotoxicity in an Iron Deficiency Rat Model
Source: Chemother Res Pract. 2014 Apr 30;2014:570241. doi: 10.1155/2014/570241 (PMC4022115; doi:10.1155/2014/570241)
Supplement: Supplementary file 1 — DOX-associated nephrotoxicity and its modulation with FCM were characterised in SHR-SP rats with IDA that was induced by low iron diet. The assessments involved the following evaluations at study end: renal function (creatinine clearance, 24 h proteinuria, urine NGAL), oxidative stress (GSH:GSSG ratio, MDA, catalase, Cu,Zn-SOD, GPx activity), histological and immunohistochemical parameters (Masson's Trichrome and Sirius Red staining, immunodetection of TNF-alpha, IL-6, iNOS, nitrotyrosine and ferritin L-chain). [file 570241.f1.pdf]

Supplemental Data for

Ferric carboxymaltose-mediated attenuation of doxorubicin-induced cardiotoxicity in an iron deficiency rat model

Jorge Eduardo Toblli [1], Carlos Rivas [1], Gabriel Cao [1], Jorge Giani [1], Felix Funk [2], Lee Mizzen [3], Fernando Dominici [1]

[1]Hospital Aleman, Laboratory of Experimental Medicine, Av. Pueyrredon 1640, 1118 Buenos Aires, Argentina (jorgetoblli@fibertel.com.ar; crivas66@gmail.com; gabrielcao@fibertel.com.ar; jorgegiani@hotmail.com; dominici@qb.ffyb.uba.ar)

[2] Vifor (International) AG, Rechenstrasse 37, CH-9001, St. Gallen, Switzerland (felix.funk@viforpharma.com)

[3] Vifor Pharma, 1201-4464 Markham St, Saanich, BC V8Z 7X8, Canada (lee.mizzen@viforpharma.com)

Short title: Iron can reduce doxorubicin-induced cardiotoxicity

Corresponding author:

Prof Jorge E. Toblli, MD, PhD
Laboratory of Experimental Medicine
Hospital Alemán, School of Medicine, University of Buenos Aires
Av. Pueyrredon 1640
1118 Buenos Aires, Argentina
Tel: +54 11 4 827 7000 (extension 2785)
Fax: +54 11 4805 6087
E-mail: jorgetoblli@fibertel.com.ar

The supplemental file includes:

Materials and Methods

Table S1. Renal function according to iron diet, DOX treatment and supplementation with FCM

Table S2. Renal oxidative stress according to iron diet, DOX treatment and supplementation with FCM

Table S3. Renal histological and immunohistochemical assessment according to iron diet, DOX treatment and supplementation with FCM

Figure S1. Immunohistochemical evaluation of nitrosative stress in the kidney

Figure S2. Immunohistochemical evaluation of inflammation in the kidney

Supplementary Methods

Modulation of DOX-induced kidney toxicity with FCM

After 3 weeks acclimatisation, 6 week old male SHR-SP (n=64) were randomised into eight groups (Groups 1-8; n=8/group). Groups 1-6 were fed LID while Groups 7 and 8 were fed ND for 7 weeks. Then, Groups 1-5 and Group 8 received 6 weekly 0.5 mL i.p injections of DOX at 4 mg/kg BW. In addition, starting at the second weekly DOX injection, Groups 1 and 2 received 5 weekly 0.5 mL intravenous (i.v.) injections of ferric carboxymaltose (FCM) at 3 mg iron/kg BW in 0.5 ml concurrent with or 3 days after DOX treatment, respectively, while Groups 3 and 4 received a single 0.5 mL i.v. injection of FCM at 15 mg iron/kg BW concurrent with or 3 days after the second DOX injection, respectively. Group 8 (fed ND) received a single i.v. injection of FCM at 15 mg iron/kg BW concurrent with the second DOX injection. As a control for multiple FCM injections, Group 5 received 5 weekly i.v. injections of saline concurrent with DOX treatment. As controls for combined treatment with DOX and FCM, Group 6 (fed LID) and Group 7 (fed ND) received 6 weekly i.p. and 5 weekly i.v. saline injections, respectively. Two weeks after the last DOX injection, all rats were anaesthetised (sodium thiopental 40 mg/kg BW) and euthanised by subtotal exsanguination. Kidneys were perfused with saline and tissue samples taken from the blood-free organ for histological and immunohistochemical assessment.

Evaluation of renal function

At the end of the study (see Methods in main manuscript for study outline), renal function tests (creatinine [Cr] clearance and 24 h proteinuria) were performed by standard techniques. Urine neutrophil gelatinase-associated lipocalin (NGAL) levels were measured in samples obtained at the end of the study using a rat NGAL ELISA Kit (BioPorto Diagnostics A/S Gentofte, Denmark).

Evaluation of oxidative stress in the kidney

Tissue samples of saline-perfused kidney cortex and medulla were homogenised (1:3, weight:volume [w:v]) in ice-cold sucrose (0.25 mol/L). Reduced glutathione (GSH) levels in supernatants after centrifugation at 10,000 xg were measured following methods as previously described [23]. Calculation of the ratio of reduced:oxidised glutathione (GSH:GSSG) was done as described [22]. Another fraction of the tissue was homogenised (1:10, w:v) in sodium phosphate buffer (0.05 mol/L, pH 7.4). One portion of the homogenate was used directly for determination of thiobarbituric acid-reactive species (TBARS) expressed as malondialdehyde (MDA), while another portion was centrifuged at 9,500 xg and 4°C for 15 min. The supernatant was used for assessment of catalase activity as described [21]. Finally, another fraction of tissue was homogenized (1:3, w:v) in ice-cold sucrose (0.25 mol/L) and centrifuged at 105,000 xg for 90 min. The supernatant was used for assessment of Cu,Zn superoxide dismutase (Cu,Zn-SOD) and glutathione peroxidase (GPx) activity as described [20].

Histological and immunohistochemical evaluation of the kidney

For histologic analysis of the kidney, perfused tissue obtained at study termination was fixed in phosphate-buffered 10% formaldehyde (pH 7.2) and embedded in paraffin. Three micron sections were cut and stained with Masson's Trichrome and Sirius Red as described [24]. Histological evaluations were performed using a light microscope, Nikon E400 (Nikon Instrument Group, Melville, NY, USA).

Immunohistochemistry of kidney tissue sections was performed with antibodies against tumor necrosis factor-α (TNF-α, AF-510-NA diluted 1:50, R&D Systems, Minneapolis, MN, USA), interleukin-6 (IL-6, sc-1265 diluted 1:100, Santa Cruz Biotechnology, Santa Cruz, CA, USA), inducible nitric oxide synthase (iNOS, sc-651 diluted 1:150, Santa Cruz Biotechnology), nitrotyrosine (AB5411, diluted 1:100, Millipore, Billerica, MA, USA), and ferritin light chain (L-

ferritin, sc-14420 diluted 1:100, Santa Cruz Biotechnology). Staining was performed with a commercially modified avidin-biotin-peroxidase complex and hematoxylin counterstaining as described [25]. Immunostaining is expressed as percentage of positive staining per area from 20 random images viewed at x400 magnification. All immunostained tissue samples were evaluated independently by two investigators blinded to sample identity. Measurements were made using an image analyzer, Image-Pro Plus version 4.5 for Windows (Media Cybernetics, LP, Silver Spring, MD, USA).

Statistical methods

Values are shown as mean±SD. All statistical analyses were performed using absolute values and processed by GraphPad Prism, version 5.01 for Windows (GraphPad Software, Inc. San Diego, CA, USA). For parameters with Gaussian distribution, comparisons among groups were carried out using ANOVA; for parameters with non-Gaussian distribution (e.g. histological data), comparisons were performed by Kruskal-Wallis test (nonparametric ANOVA) and Dunn´s multiple comparison test. A p-value <0.05 was considered significant.

Table S1: Renal function according to iron diet, DOX treatment and supplementation with FCM

Group	1	2	3	4	5	6	7	8
**Parameter**								
Cr clearance (mL/min)	3.8±0.1	3.8±0.2	3.8±0.2	3.8±0.1	3.7±0.1	3.8±0.2	4.0±0.2	2.9±0.2*
NGAL (µg/ml)	2.1±0.5	1.8±0.4	1.9±0.5	0.8±0.4†	4.8±0.3*	1.3±0.2†	0.5±0.1†‡	6.4±0.4*
Proteinuria (mg/day)	59.3±4.1§	52.9±4.3§	53.8±3..8§	50.6±5.7§	79.9±6.0ǁ	48.3±5.6§	36.4±3.5*	123.5±8.5

Data presented as mean±SD. Cr, creatinine; NGAL, neutrophil gelatinase-associated lipocalin.

* p< 0.01 vs. all groups

† p< 0.01 vs. G1,G2 and G3

‡ p< 0.01 vs. G6

§ p< 0.01 vs. G5 and G8

ǁ p< 0.01 vs. G8

Table S2: Renal oxidative stress according to iron diet, DOX treatment and supplementation with FCM

Group	1	2	3	4	5	6	7	8
**Parameter**								
MDA (nmol/mg protein)	3.9±0.2*	3.8±0.3*	3.1±0.2†	2.8±0.2‡	4.6±0.3§	2.7±0.1‖	2.1±0.2¶	5.9±0.2
CuZn-SOD (U/mg protein)	24.1±1.4*	22.3±1.9*	18.2±1.7†	16.7±1.6‡	28.8±1.8§	14.0±1.1‖	9.9±1.0¶	53.5±3.7
GPx (U/mg protein)	449±23*	443±20*	406±18†	402±21‡	489±22§	369±25‖	270±21¶	542±31
GSH:GSSG ratio	3.4±0.2*	3.5±0.2*	3.8±0.1†	4.0±0.1‡	2.5±0.1§	4.1±0.2‖	4.6±0.2¶	1.9±0.3

Data presented as mean±SD. MDA, malondialdehyde; CuZn-SOD, copper-zinc superoxide dismutase; GPx, glutathione peroxidase; GSH:GSSG, reduced:oxidized glutathione.

* p<0.01 vs. G3 to G8

† p<0.01 vs. G5 to G8

‡ p<0.01 vs. G5, G7 and G8

§ p<0.01 vs. G6 to G8

‖ p<0.01 vs. G7 to G8

¶ p<0.01 vs. G8

Table S3: Renal histological and immunohistochemical assessment according to iron diet, DOX treatment and supplementation with FCM

Group	1	2	3	4	5	6	7	8
**Parameter**								
Sirius Red (%/area)	7.2±1.2	7.1±1.2	7.1±1.0	7.0±1.2	15.1±1.7*	10.6±1.3*	6.6±1.1	17.9±1.8*
Nitrotyrosine (%/area)	19.5±2.1	18.4±2.7	16.1±2.4	15.6±1.6ǁ	23.1±3.6*	16.3±2.0	11.1±1.5*	29.8±2.5*
L-ferritin (%/area)	11.5±1.3	11.6±1.8	11.9±1.6	12.1±1.5	2.3±0.4†	2.2±0.4†	4.7±0.9*	15.8±1.2*
TNF-α (%/area)	7.1±0.7	7.3±0.7	7.3±0.8	7.1±1.0	15.5±1.4‡	7.8±1.1	4.9±0.6*	16.4±1.3‡
IL-6 (%/area)	10.5±1.6	10.6±1.6	10.3±1.7	10.4±1.1	27.3±2.2‡	10.7±0.9	5.8±1.0*	25.7±2.9‡
iNOS (%/area)	4.6±0.4	4.2±0.7	4.3±0.7	4.4±0.4	13.5±1.2§	9.5±1.1*	4.5±0.9	14.9±1.2§

Data presented as mean±SD. L-ferritin, ferritin light chain; TNF-α, tumor necrosis factor-alpha; IL-6, interleukin 6; iNOS, inducible nitrogen oxide synthase.

* p<0.01 vs. all groups

† p<0.01 vs. G1, G2, G3 and G4

‡ p<0.01 vs. G1, G2, G3, G4 and G6

§ p<0.01 vs. G1, G2, G3, G4, G6 and G7

ǁ p<0.05 vs. G1

Figure Legends

Figure S1. Immunohistochemical evaluation of nitrosative stress in the kidney. Representative light micrographs of kidney sections from Groups 1-8 (G1-G8) immunostained with antibodies against nitrotyrosine (panel A) or iNOS (panel B) and counterstained with hematoxylin. The greatest level of nitrotyrosine immunostaining is seen in kidney sections from ND-fed rats treated with DOX and FCM (G8), followed by LID-fed rats treated with DOX (G5) and LID-fed rats treated with saline (G6). In LID-fed rats treated with DOX and FCM (G1-G4), staining was lower compared to G5, whereas only low levels of staining were seen in LID-fed rats treated with saline (G7). Similar Group trends were observed with iNOS immunostaining. Original magnification, x400.

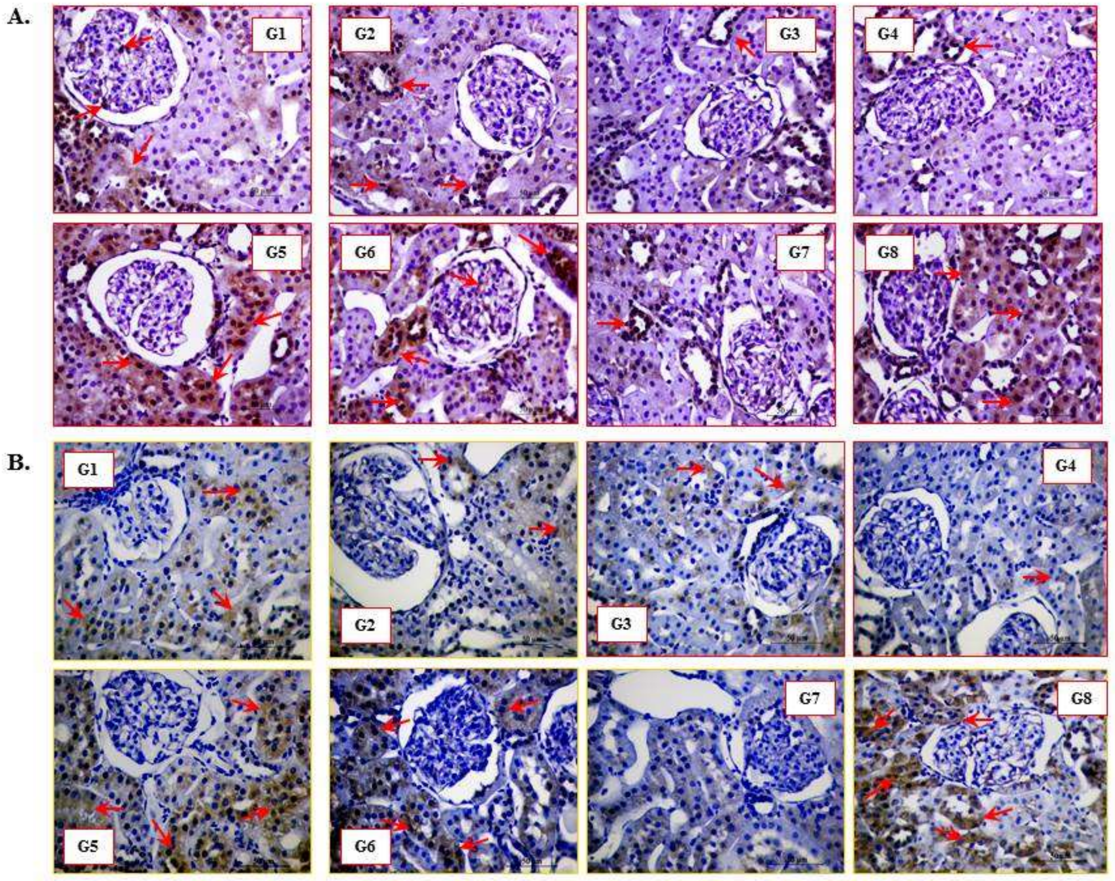

Figure S2. Immunohistochemical evaluation of inflammation in the kidney. Representative light micrographs of kidney sections from Groups 1-8 (G1-G8) immunostained with antibodies against TNF-α (panel A) or IL-6 (panel B) and counterstained with hematoxylin. Significant proinflammatory cytokine immunostaining is seen in kidney sections from ND-fed rats treated with DOX and FCM (G8) and in LID-fed rats treated with DOX (G5). Lowest degree of staining is seen in ND-fed rats treated with saline (G7). In LID-fed rats treated with DOX and FCM (G1-G4), a moderate level of staining is observed similar to that seen in LID-fed rats treated with DOX only (G6). Original magnification, x400.

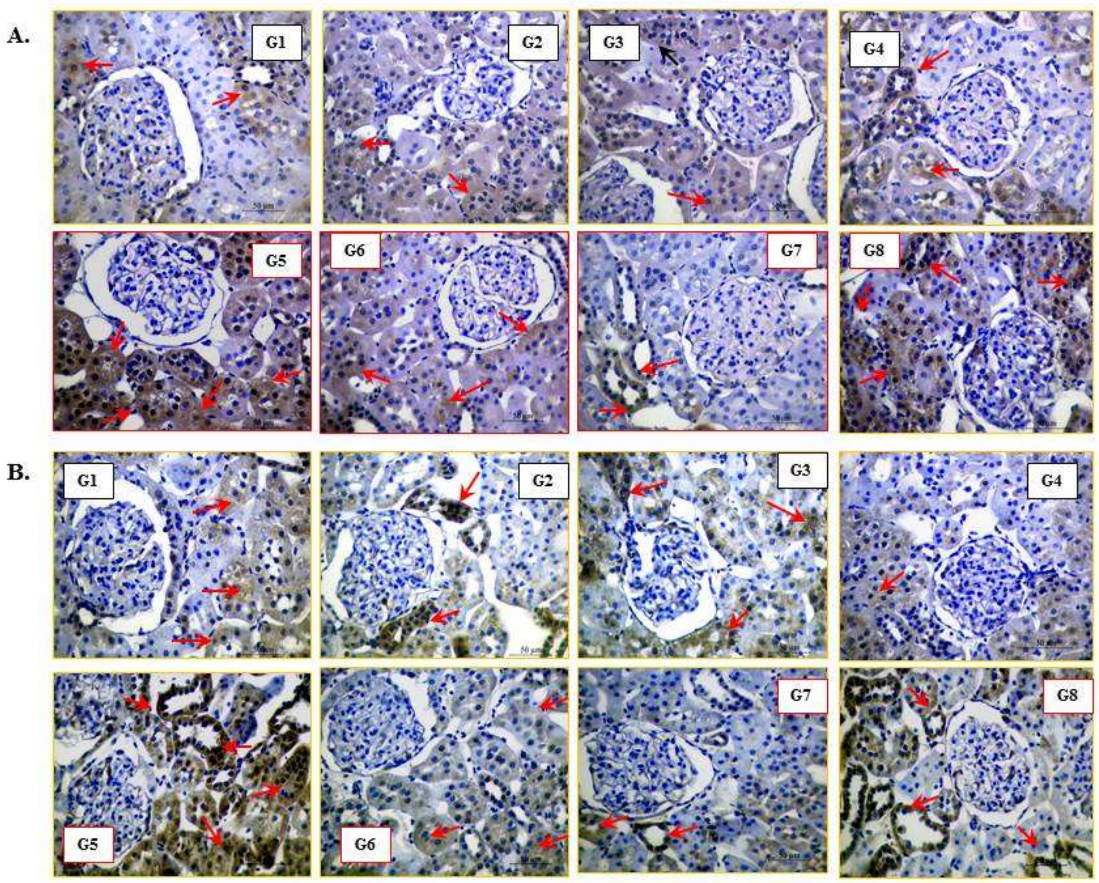

References

1. B. Chance, A. Maehly, "Assay of catalase and peroxidase," In *Methods in enzymology*, edited by S. P. Colowick, N. O. Kaplan, New York, Academic Press, pp. 764-768, 1955.

2. J. B. Owen, D. A. Butterfield, "Measurement of oxidized/reduced glutathione ratio," *Methods Mol Biol*, vol. 648, pp. 269-277, 2010.

3. J. E. Toblli, G. Cao, J. F. Giani, M. C. Munoz, M. Angerosa, and F. P. Dominici, "Long-term treatment with nebivolol attenuates renal damage in Zucker diabetic fatty rats," *J Hypertens*, vol. 29, no. 8, pp. 1613-1623, 2011.

4. J. E. Toblli, G. Cao, L. Oliveri, and M. Angerosa, "Differences between original intravenous iron sucrose and iron sucrose similar preparations," *Drug Res*, vol. 59, no. 4, pp. 176-190, 2009.

5. M. L. Schuman, M. S. Landa, J. E. Toblli et al., "Cardiac thyrotropin-releasing hormone mediates left ventricular hypertrophy in spontaneously hypertensive rats," *Hypertension*, vol. 57, no. 1, pp. 103-109, 2011.

6. J. E. Toblli, G. Cao, C. Rivas, and H. Kulaksiz, "Heart and iron deficiency anaemia in rats with renal insufficiency: the role of hepcidin," *Nephrology (Carlton)*, vol. 13, no. 7, pp. 636-645, 2008.
